# Awareness, attitudes, and practices related to the swine influenza pandemic among the Saudi public

**DOI:** 10.1186/1471-2334-10-42

**Published:** 2010-02-28

**Authors:** Hanan H Balkhy, Mostafa A Abolfotouh, Rawabi H Al-Hathlool, Mohammad A Al-Jumah

**Affiliations:** 1 King Abdullah International Medical Research Center (KAIMRC), King Saud bin Abdulaziz University for Health Sciences (KSAU-HS), Riyadh, Kingdom of Saudi Arabia

## Abstract

**Background:**

During an infectious disease outbreak, it is critical to learn as much as possible about the concerns, knowledge, attitudes, and behavior of the public. Such information can be crucial to the improvement of communication efforts by public health officials and clinicians. The aim of this study was to identify awareness, attitudes, and practices related to influenza A (H1N1) among the Saudi public.

**Methods:**

A cross-sectional study of 1,548 adult subjects recruited from various shopping malls in Riyadh and Jeddah was conducted. All of the subjects were interviewed using a questionnaire that tested their knowledge, attitudes, and use of precautionary measures in relation to the H1N1 influenza pandemic.

**Results:**

More than half (54.3%, 840/1548) of the participants showed high concern, 43.7%(677/1548) showed a low level of knowledge, and 60.8%(941/1548) had taken minimal or no precautionary measures. After adjusting for other variables, education level was the only significant predictor of the level of concern (p < 0.001), while greater precautionary measures were taken by participants who were male (p < 0.001), older (p = 0.047), better educated (p = 0.04), and more knowledgeable (p < 0.001). More than one-third (38.3%) of participants were not convinced that the MOH reports about the disease were true, and only 16.1% of the participants reported receiving information from health providers.

**Conclusions:**

High concern did not translate into a higher compliance with precautionary recommendations, possibly due to the low level of knowledge about the disease among the public. Frequent communication between physicians and the public is recommended to help dispel myths about the disease and to spread better information about the role that the public can play in limiting the spread of the disease.

## Background

A novel influenza A (H1N1) or Swine flu has recently emerged from Mexico causing the first pandemic of the century [1,2]. It has been known from previous experiences that anxiety and misconceptions of infectious outbreaks, whether natural or terrorist born, may lead to unnecessary worry and chaos in a situation where the public is threatened [3,4]. Further, misconceptions and worries have led to inappropriate behaviour by the public such as; refusal to comply with precautionary measures, including wearing a mask or accepting a vaccination; avoidance of certain activities including visiting the hospital due to fear of healthcare facilities as a venue for aqcuiring the infection [5-7]. Understanding the perception of the public and their potential resources to infectious disease threats would assist public health agencies to pinpoint knowledge gaps which may be utilized in developing educational programs to increase the awareness of the public.

The Kingdom of Saudi Arabia (KSA) has been very transparent in its activities, announcing early cases of swine flu and setting in place a strict method for surveillance. The country hosts more than 3 million pilgrims and visitors to the two holy mosques every year, and it is therefore especially important to develop a clear plan for educating and preparing the public and pilgrims for a potential infectious crisis. The study started after the WHO raised its pandemic alert status to phase 6. At that time, mitigation measures in Saudi Arabia focused on identifying, treating, and isolating people who had the disease or were exposed to individuals who had the disease.

Two major resources on the developments of the H1N1 pandemic existed for the Saudi public. The media, including both televised and written media, and to a lesser extent the radio; the second was the internet, where free uncensored writing and U-tube postings take place. There was a large need for the dissemination of accurate information to overcome the misinformed dialogue taking place on TV, newspapers and internet. During the early days of the pandemic, and for the initial announcement of the first case of confirmed H1N1 within the Kingdom, there was clear communication from the MOH on the case and its developments. The Minister of Health, soon thereafter, developed a National Scientific Committee to deal with all rising concerns on the pandemic, including at the time: medication needs, vaccination needs, vaccination prioritization and defining high risk groups, school suspension policies and identifying clear routes for communication with the Ministry of Education. The scientific committee was able to provide educational material, such as brochures, pamphlets and stands, on the signs and symptoms of H1N1for dissemination through shopping malls, mosques, airports and schools. The members of the scientific committee were also requested to provide scientific statements on the developments of the pandemic locally and internationally.

Learning more about the concerns, knowledge, attitudes, and behaviors of the public during an infectious disease outbreak can be crucial to improve communication efforts by public health officials and clinicians. To the best of our knowledge, this is the first study from the Kingdom of Saudi Arabia that focuses on understanding the public's awareness of and attitudes toward a pandemic threat.

## Methods

### Study Subjects

Adults over the age of 18 years (both male and female) in shopping malls in the cities of Riyadh and Jeddah who provided verbal consent, were eligible to participate in the survey. Non-Saudi subjects and those non-willing to participate were excluded.

### Study Design

A cross-sectional study design was used.

### Study Population and Sampling Technique

Riyadh and Jeddah were selected, being the two largest cities in Saudi Arabia, to ensure good representation from across the country. A number of large malls that serve different geographical areas of both cities were identified. Proportional quota sampling was used to ensure that respondents were demographically representative of the general population with quotas based on age, sex, work status, region and social class. All adults shopping in these malls within two weeks between 8 and 22 September, 2009. who were willing to participate in the study were interviewed. Each data collector spent an average of 3 hours in each mall at randomly chosen time of the day to recruit participants. Of 1,601 possible participants, 1,548 subjects of both sexes were successfully interviewed (response rate = 97%).

### Data Collection Methods

An interview questionnaire was designed to collect the following data:

a) Socio-demographic characteristics such as gender, age, education, and occupation.

b) Knowledge about the disease, its nature, mode of transmission, symptoms and signs, incubation period, period of communicability, and preventive measures. This knowledge was assessed by 17 factual statements that participants responded to with "yes" or "no." A scoring system was applied to assess the level of knowledge of each subject: 1 point was given for each correct answer, and 0 point was given for each incorrect answer. Participants were grouped into three categories according to their level of knowledge: low (<10 points), average (10-12 points), and high (13 or more points).

c) Attitudes toward and perceptions of the disease, its severity, governmental efforts to combat it, and disease outcomes were assessed by six attitudinal statements that participants responded to with "strongly agree," "agree," "neutral," "disagree," or "strongly disagree." A scoring system was applied using the Likert 5-point scale; 5 points were assigned to "strongly agree," and 1 point was assigned to "strongly disagree." Negative attitude statements were scored from 1 (for those who strongly agreed) to 5 (for those who strongly disagreed). Thus, the total attitude score ranged from 6 to 30 points. For each statement, the participant was considered extremely concerned if he/she agreed or strongly agreed. Subjects were grouped into three categories according to their level of concern: extremely concerned (if agreement was evident for 5-6 statements), quite concerned (if agreement was evident for 3-4 statements), and little concerned (if agreement was evident for 2 or fewer statements).

d) Each participant was asked to report the precautionary measures that s/he has been using during the epidemic to prevent infection. Participants' responses were assessed in accordance with the six precautionary measures recommended by the U.S. Center for Disease Control (CDC). A scoring system was applied in which each participant was given 1 point for each precautionary measure taken. Thus, the total precaution score ranged from 0 to 6 points. A high level of precaution was considered to be 5-6 points, a moderate level was 3-4 points, and a poor level was 2 points or less.

Research coordinators and research assistants at the King Abdullah International Medical Research Center (KAIMRC) were trained to conduct the interviews. Each day, one of the co-authors assessed the accuracy and completeness of the data collection forms and the standardization of the procedures.

### Data Analysis

Data entry and statistical analysis were performed with the Statistical Package for Social Science (SPSS) software program for Windows (version 17.0). Descriptive statistics, such as percentages, means, and standard deviations, were calculated. For categorical data, a chi-square test was applied. For continuous data, both *Student's t*-test and *ANOVA *were applied. Multiple regression analyses were performed to determine the significant predictors of both the level of concern and the level of precaution. Statistical significance was considered at p < 0.05 for all analyses.

### Ethical Considerations

The study protocol (Application # *RR09/024*) received ethical approval from the Institutional Review Board (IRB) of the National Guard Health Affairs (NGHA), Riyadh, Saudi Arabia.

## Results

### Participant Demographic Characteristics (Table 1)

**Table 1:** Demographic Characteristics of the study sample

Characteristics	Male(n = 828)		Female (n = 720)		Total (n = 1548)	
	
	No.	%	No.	%	No.	%
Age group:						

18-24 yr.	246	29.7	261	36.3	507	32.8

25-39 yr.	455	55.0	368	51.1	823	53.2

40-59 yr.	118	14.3	87	12.1	205	13.2

60 yr. or more	9	1.1	4	0.6	13	0.8

	χ^2 ^= 8.759, p = 0.033					

Marital Status:						

Single	397	47.9	291	40.5	688	44.5

Married	416	50.2	396	55.1	812	52.5

Widow	2	0.2	11	1.5	13	0.8

Divorced	13	1.6	21	2.9	34	2.2

	χ^2 ^= 17.343, p < 0.001					

Education:						

Non-educated	17	2.1	17	2.4	34	2.2

Less than secondary	56	6.8	70	9.7	126	8.2

Secondary	230	27.8	198	27.6	428	27.7

University	461	55.7	413	57.5	874	56.5

Higher	64	7.7	20	2.8	84	5.4

	χ^2 ^= 21.916, p < 0.001					

Employment status						

At work	671	81.7	280	39.2	951	62.0

Non-working	150	18.3	434	60.8	584	38.0

	χ^2 ^= 292.832,p < 0.001					

A total of 1,548 interviews were conducted (828 males and 720 females). Most of the participants were in the age groups of 18-24 years (53.2%) and 25-39 years (32.8%). About one-half of the participants were married (52.5%), and the majority had completed their secondary education (89.6%). Nearly two-thirds (62%) of the subjects were employed. Compared to the male participants, female participants were significantly younger (p = 0.033), less educated (p < 0.001), and more likely to be married (p < 0.001) and unemployed (p < 0.001).

### Knowledge Assessment (Table 2)

**Table 2:** Knowledge about influenza (H1N1) among the Saudi Public:

	Statement	Yes	No	Don't know
		
		%	%	%
	***The cause of swine flu is ...***			

**1**	Virus.	95.4*	2.1	2.5

**2**	Immunodeficiency.	27.6	61.0*	11.4

**3**	Inherited disease.	4.3	88.2*	7.5

**4**	Swine flu is a communicable disease	92.3*	3.5	4.2

	***Swine flu is transmitted through ...***			

**5**	Droplets after coughing or sneezing.	95.5*	2.8	1.7

**6**	Touching the infected person.	61.0*	31.9	7.0

**7**	The use of objects used by an infected person.	73.1*	19.1	7.8

**8**	Sexual route.	42.8	37.2*	20.0

	***What are the symptoms and signs of swine flu?***			

**9**	Same as seasonal flu (fever, cough, sore throat, muscle ache, etc.)	93.8*	3.4	2.8

**10**	Swine flu may lead to death immediately	11.4*	77.1	11.4

**11**	Causes the patient to look like a pig	2.3	87.7*	10.0

**12**	Swine flu can be transmitted from humans to pigs and vice versa	34.3	34.8*	30.9

**13**	Swine flu can be transmitted to humans from animals other than pigs	21.3*	41.7	37.1

**14**	There is a vaccine for swine flu	47.1	29.*	41.9

**15**	Swine flu can affect people more than once in life	29.5*	28.7	41.9

**16**	How long does it take after exposure to the cause of the disease for symptoms to appear?^@^	36.5*	62.7	0.8

**17**	After how many days can an ill person communicate with others after cure?^@^	20.1*	79.2	0.7

*... Correct answers

^@^..... Correct answers to this question were counted under the column "yes"

The majority of the participants (95.4%) were aware that the disease was a viral illness; however, a large number also mistakenly believed that the disease was an immunodeficiency disease (27.6%). Most reported accurate information about the mode of transmission, although 43% stated that sexual contact was a mode of transmission. Most participants (94%) agreed that the symptoms were the same as those of seasonal flu, although 11% of participants assumed that this illness could cause immediate death. The majority of participants were not knowledgeable about the incubation period or the period of communicability (63.5% and 80%, respectively). Nearly one-half (47%) of participants thought that there was a vaccine available for the disease at the time of the survey, though it was not yet available.

**Figure 1:**
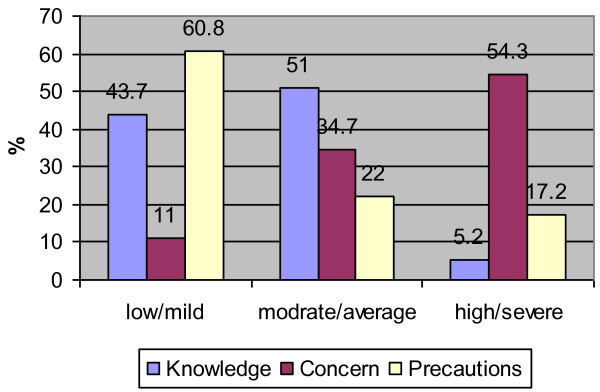
**Levels of knowledge, concern and precautions on swine flu among members of the Saudi public**.

Only 38.3% of the participants believed that the government was reporting the real number of cases, while 55.6% believed that there was underreporting of the actual number of deaths. Figure 1 shows that 44% of all participants had low knowledge about the disease; only 5.2% showed a high level of knowledge, and there was no difference between men and women (χ^2^= 1.33, p = 0.52).

### Sources of Information

The majority (84.2%) of the participants received their information about swine flu from the television; 51.1% received information from written media such as newspapers and magazines, while 48.2% received information from the internet. Only 16.1% received their information from a physician or a health educator.

### Level of Concern

The majority of participants agreed that the government should isolate patients with swine flu in special hospitals (82.4%), avoid inviting workers from areas where the disease is prevalent (87.6%), restrict travel to and from such areas (76.4%), and be ready to close schools if the number of cases increases dramatically (77.7%). More than one-half of the participants preferred not to travel during the epidemic (58.5%) and to stay at home (51.6%) (see Table 3).

**Table 3:** Responses (%) of participants to concern statements and self-reported preacautionary measures against H1N1 Influenza.

Statement	**No**.	%	95% CI
**Concern**			

We should avoid leaving our homes nowadays.	798	51.6	49.1-54.1

If I decide to travel, swine flu may prevent me.	1,029	58.5	56.0-61.0

The government should restrict travel from and to the areas of the disease.	1,181	76.4	74.3-78.5

The government should isolate patients with swine flu in special hospitals.	1,276	82.4	80.5-84.3

The government should avoid inviting workers from areas where disease is frequent.	1,353	87.6	86.0-89.2

The government should be ready to close schools if the number of cases increases dramatically.	1,202	77.7	75.6-79.8

**Precaution**			

Wash my hands often.	893	57.7	55.2-60.2

Avoid touching my eyes, nose, or mouth.	567	36.6	34.2-39.0

Cover my nose and mouth with a tissue when I cough or sneeze.	588	38.0	35.6-40.4

I throw the tissue in the trash after I use it.	417	26.9	24.7-29.1

I use a face mask in crowded areas.	870	56.2	53.7-58.7

If I have a flu symptoms, I avoid normal activities, including work, school, travel, shopping, etc..	398	25.7	23.5-27.9

Figure 1 shows that high concern was prevalent in more than one-half (54.3%) of all participants, with no differences between men and women (χ^2^= 1.68, p = 0.64).

### Precautionary Measures

More the one-half of the participants reported frequent hand washing (57.7%) and the use of a facemask in crowded areas (56.2%). Moreover, one-third reported avoiding touching their eyes, nose, or mouth (36.6%) and covering their nose and mouth with a tissue when coughing or sneezing (38.0%). However, only one-fourth of participants reported throwing the tissue in the trash after use (26.9%) and avoiding normal activities if they have flu-like symptoms (25.7%) (see Table 3). About two-thirds of all participants (60.8%) reported not taking minimal or mild precautions to prevent infection, and only 17.2% reported a high level of precautions (Figure 1).

Table 4 shows multiple regression analyses of the concern scores and the precaution scores in relation to several independent variables. Education was the only significant predictor of concern, and the level of education was inversely related to the degree of concern (p < 0.001). On the other hand, significant predictors of precautionary scores included age, gender, education, and the level of knowledge. A high level of precaution was taken by males (p < 0.001), older individuals (p = 0.047), those with a higher education (p = 0.04), and those with a higher level of knowledge about H1N1 (p < 0.001).

**Table 4:** Multiple regression analysis of degree of concern (score) and precautionary measures (score) on some independent predictors.

Independent predictors		B	SE	t-value	p-value
**Age**	Concern	0.11	0.18	0.64	0.52

	Precaution	0.14	0.07	1.99	0.047**

**Gender (female = 1)**	Concern	0.25	0.25	0.99	0.32

	Precaution	-0.39	0.1	-3.72	<0.001**

**Marital status (single = 1)**	Concern	0.07	0.24	0.27	0.79

	Precaution	0.06	0.1	0.55	0.59

**Education**	Concern	-0.49	0.15	-3.29	0.001**

	Precaution	0.13	0.06	2.06	0.04**

**Employment (employed = 1)**	Concern	0.2	0.28	0.74	0.46

	Precaution	0.18	0.11	1.63	0.1

**Knowledge (score)**	Concern	-0.02	0.06	-0.27	0.79

	Precaution	0.09	0.03	3.84	<0.001**

**..... Statistically significant.

## Discussion

Novel influenza A (H1N1), also known as swine flu, has recently emerged from Mexico and has caused the first pandemic of the century [1]. If people are to respond appropriately during an outbreak of infectious disease, they need to have some basic knowledge about disease transmission, availability of vaccines and effective medical treatment. Many reports have examined the various levels of knowledge about infectious agents and public behavior in relation to these infections; such studies have primarily focused on the SARS and avian influenza outbreaks [5,8]. Other studies have been recently published specifically on behavioral and attitudinal responses to pandemic (H1N1) 2009 Influenza [7,9-11].

In a previous study designed to assess the implications of public understanding of avian influenza, researchers found that the majority of participants did not believe in a pandemic and believed that dealing with the disease was the responsibility of the government [12]. Opinions about the credibility of health information varied from distrust to belief in the credibility of information released by the local health department. In our study, only 38.3% of the participants believed that the government was reporting the real number of H1N1 cases, and 55.6% believed that the number of cases was being underreported; these findings might reflect some distrust in the announcements of the Ministry of Health. However, it may have related to realism by the public that not all cases could be detected.

If people are to respond appropriately during an outbreak of infectious disease, they need to have some basic knowledge about how the disease is spread and whether there is a vaccine against the disease or an effective medical treatment that can be administered once someone contracts it. Many reports have highlighted various levels of knowledge towards infectious agents and the public behaviour towards these infections, especially after the SARS and avian influenza outbreaks [5,12]. In the present study, 44% of participants had low knowledge, with only 5% having high level of knowledge. Low knowledge was evident with regard to the period of communicability (20.1%) and the incubation period (36.5%) of Swine flu.

In a recent review of behavioral responses to influenza pandemics in the 20^th ^century [13], the only two measures that had strong support by scientific literature to lessen the spread of the diseases were hand hygiene and respiratory etiquette. School closure and screening of travelers had legal and ethical consequences when implemented. While the other four measures, including isolation and wearing of a surgical mask or an N95 mask, had cost effectiveness concerns and would be difficult to implement over long periods of time [14,15]. In the present study, washing hands and the use of face mask in crowded areas were the reported measures by more than a half of the participants, while other measures were less frequently reported.

It is important to know what proportion of the population is concerned about contracting a disease since those who are concerned would be expected to take more precautions. In a telephone-assisted survey of 2,081 adults above the age of 16 years, the New South Wales Department of Health found that only 48.3% of those interviewed were willing to comply with precautionary measures [16,17]. In the present study, about two-thirds of all participants (60.8%) reported either not taking any precaution, or taking minimal or mild precautions to prevent infection, with only 17% who reported high level of precautions. The frequency with which participants took high precautionary measures was significantly higher for males, older participants, those with a higher level of education, and those with a higher level of knowledge about H1N1.

In a study from Hong Kong that examined precautionary measures taken for a SARS threat, young, less-educated males were the least likely to take precautionary measures [18]. These findings differed from the results presented by Di Giuseppe, who found that those with a higher perception of risk had a lower level of education and a lower socio-economic level but were more likely to comply with precautionary measures that would limit the spread of the disease [19].

Should we worry? The word "pandemic" alone and the announcement that we are at Phase 6 gives an impression that there are many people dying every day; this impression may cause people to worry, to empty classrooms, to fill emergency rooms, and to disrupt normal business and economic activities. The general public should know that swine flu is global but not severe and that the flu pandemic is not defined by its severity. In the present study, high concern was prevalent in more than one-half (54%) of all participants, although it took different forms. The majority of participants agreed that the government should isolate patients with Swine flu in special hospitals, avoid inviting workers from areas where the disease is prevalent, restrict travel to and from such areas, and be ready to close schools if number of cases dramatically increases. More than one-half of participants prefereed not to travel during epidemic and to stay at home.

Regional differences in anxiety towards influenza A, H1N1, have been suggested [11]. This might explain the differences and similarities in the results of the different studies. The findings of the present study were similar to those of a study conducted in 2005 on precautionary measures using a hypothetical influenza pandemic, where 5 european countries and 3 asian regions with 3,436 participants were involved [20]. However, these findings were not in agreement with those of two previous studies, one in the United Kingdom [7], and the other in Australia [10] where low levels of anxiety towards swine flu were detected. Education in the present study was the only significant predictor of concern, where the level of education was inversely related to the level of concern (in terms of concern score),while employment was the only individual variable to affect inmplementing precautionary measures in a previous study [20]. In another study, the higher level of threat perception was among the elderly, those with poor self-rated health, those lacking formal qualifications, those with low household incomes, and those living in rural areas [18].

## Conclusions

There has been a strong call for public health officials to prepare for the influenza pandemic, while there has been very little focus on how to prepare the public for such a catastrophe. Although it has been suggested that culture and individual anxiety are important predictors of behavioral responses to pandemic influenza [11], the present study revealed a high level of concern that did not translate into a higher compliance with precautionary measures. This may be explained by a poor understanding of the disease and its transmission [21]. Meanwhile, the disbelief in the government reports about the outbreak could have negative implications for compliance with official advice. Misconceptions about the disease may be related to the use of television and newspapers (which highlight reported cases and deaths) as the primary sources of information on swine flu.

Our study found that physicians and health educators seem to play an insufficient role in the education of the public. Perhaps increased communication between physicians and the public would help dispel myths about the disease and help spread accurate information about the role that the public can play in limiting the spread of the disease. Collaborative efforts orchestrated by the MOH are needed and should focus on public education and training through media resources.

## Competing interests

The authors declare that they have no competing interests.

## Authors' contributions

All authors contributed to the design and execution of the study and analyses.

HHB & MAA were actively involved in writing the manuscript. MAJ commented on drafts presented to him. All authors read and approved the final manuscript.

## Pre-publication history

The pre-publication history for this paper can be accessed here:

http://www.biomedcentral.com/1471-2334/10/42/prepub

## References

[B1] Scalera NM Mossad SB The first pandemic of the 21st century: a review of the 2009 pandemic variant influenza A (H1N1) virus Postgrad Med 2009 121 5 43 7 10.3810/pgm.2009.09.2051 19820273

[B2] Machado AA How to prevent, recognize and diagnose infection with the swine-origin Influenza A (H1N1) virus in humans J Bras Pneumol 2009 35 5 464 9 10.1590/S1806-37132009000500013 19547857

[B3] Blendon RJ Benson JM DesRoches CM Raleigh E Taylor-Clark K The public's response to severe acute respiratory syndrome in Toronto and the United States Clin Infect Dis 2004 38 7 925 31 10.1086/382355 15034821

[B4] Barbera J Macintyre A Gostin L Inglesby T O'Toole T DeAtley C Tonat K Layton M Large-scale quarantine following biological terrorism in the United States: scientific examination, logistic and legal limits, and possible consequences JAMA 2001 286 21 2711 7 10.1001/jama.286.21.2711 11730447

[B5] Tang CS Wong CY An outbreak of the severe acute respiratory syndrome: predictors of health behaviors and effect of community prevention measures in Hong Kong, China Am J Public Health 2003 93 11 1887 8 10.2105/AJPH.93.11.1887 14600058 PMC1448068

[B6] Tang CS Wong CY Factors influencing the wearing of facemasks to prevent the severe acute respiratory syndrome among adult Chinese in Hong Kong Prev Med 2004 39 6 1187 93 10.1016/j.ypmed.2004.04.032 15539054 PMC7133369

[B7] Rubin GJ Amlot R Page L Wessely S Public perceptions, anxiety, and behaviour change in relation to the swine flu outbreak: cross sectional telephone survey BMJ 2009 339 b2651 10.1136/bmj.b2651 19574308 PMC2714687

[B8] de Zwart O Veldhuijzen IK Elam G Aro AR Abraham T Bishop GD Richardus JH Brug J Avian influenza risk perception, Europe and Asia Emerg Infect Dis 2007 13 2 290 3 10.3201/eid1302.060303 17479894 PMC2725846

[B9] Eastwood K Durrheim DN Jones A Butler M Acceptance of pandemic (H1N1) 2009 influenza vaccination by the Australian public MJA 2010 192 1 33 36 20047546 10.5694/j.1326-5377.2010.tb03399.x

[B10] Seale H McLaws ML Heywood AE Ward KF Lowbridge CP Van D Gralton J MacIntyre CR The community's attitude towards swine flu and pandemic influenza Med J Aust 2009 191 5 267 9 19740048 10.5694/j.1326-5377.2009.tb02781.x

[B11] Goodwin R Haque S Neto F Myers LB Initial psychological responses to Influenza A, H1N1 ("Swine flu") BMC Infectious Diseases 2009 9 166 10.1186/1471-2334-9-166 19807908 PMC2765446

[B12] Elledge BL Brand M Regens JL Boatright DT Implications of public understanding of avian influenza for fostering effective risk communication Health Promot Pract 2008 9 4 Suppl 54S 9S 10.1177/1524839908319089 18936260

[B13] Balinska M Rizzo C Behavioural responses to influenza pandemics: what do we know? PLoS Curr Influenza 2009 RRN1037 10.1371/currents.RRN1037 PMC2762764 20025201

[B14] Markel H Lipman HB Navarro JA Sloan A Michalsen JR Stern AM Nonpharmaceutical interventions implemented by US cities during the 1918-1919 influenza pandemic JAMA 2007 298 6 644 54 10.1001/jama.298.6.644 17684187

[B15] Aledort JE Lurie N Wasserman J Bozzette SA Non-pharmaceutical public health interventions for pandemic influenza: an evaluation of the evidence base BMC Public Health 2007 7 208 10.1186/1471-2458-7-208 17697389 PMC2040158

[B16] Barr M Raphael B Taylor M Stevens G Jorm L Giffin M Pandemic influenza in Australia: using telephone surveys to measure perceptions of threat and willingness to comply BMC Infect Dis 2008 8 117 10.1186/1471-2334-8-117 18793441 PMC2556339

[B17] NSW Department of Health H1N1 influenza 09 (human swine influenza) factsheet http://www.healthinsite.gov.au/content/external/page.cfm?ObjID=F4A3023E-989A-3B14-A3987FE9A729ADF0&PID=67002

[B18] Leung GM Lam TH Ho LM Ho SY Chan BH Wong IO The impact of community psychological responses on outbreak control for severe acute respiratory syndrome in Hong Kong J Epidemiol Community Health 2003 57 11 857 63 10.1136/jech.57.11.857 14600110 PMC1732323

[B19] Di Giuseppe G Abbate R Albano L Marinelli P Angelillo IF A survey of knowledge, attitudes and practices towards avian influenza in an adult population of Italy BMC Infect Dis 2008 8 36 10.1186/1471-2334-8-36 18366644 PMC2292195

[B20] Sadique MZ Edmunds WJ Smith RD Meerding WJ de Zwart O Brug J Precautionary behavior in response to perceived threat of pandemic influenza Emerg Infect Dis 2007 13 9 1307 13 18252100 10.3201/eid1309.070372 PMC2857294

[B21] Leavitt JW Public resistance or cooperation? A tale of smallpox in two cities Biosecur Bioterror 2003 1 3 185 92 10.1089/153871303769201833 15040196

